# Efficacy of disinfectants on control and clinical bacteria strains at a zonal referral hospital in Mwanza, Tanzania: a cross sectional hospital-based study

**DOI:** 10.1038/s41598-023-45228-7

**Published:** 2023-10-21

**Authors:** Prisca Damiano, Vitus Silago, Helmut A. Nyawale, Martha F. Mushi, Mariam M. Mirambo, Emmanuel E. Kimaro, Stephen E. Mshana

**Affiliations:** 1 Department of Microbiology and Immunology, Weill Bugando School of Medicine, Catholic University of Health and Allied Sciences, P. O. Box 1464, Mwanza, Tanzania; 2 Department of Pharmaceutical Sciences, School of Pharmacy, Catholic University of Health and Allied Sciences, P. O. Box 1464, Mwanza, Tanzania

**Keywords:** Antimicrobials, Applied microbiology, Infectious-disease diagnostics, Immunology, Microbiology, Molecular biology

## Abstract

Contaminated-hospital surfaces are an important source of pathogenic bacteria causing health-care associated infection (HCAIs). Monitoring the performance of disinfectants that are routinely used to clean hospital surfaces is critical for prevention and control of HCAIs. Nevertheless, the evaluation of the performance of disinfectants and their efficacy are not routinely practiced in most resource-limited countries. This study was designed to determine the efficacy of sodium dichloroisocyanurate (NaDCC) and chloroxylenol against American Type Culture Collection (ATCC) and their respective multidrug resistant (MDR) strains causing neonatal sepsis at a zonal referral hospital in Mwanza, Tanzania. Four ATCC (n = 4) and their respective MDR strains of *Klebsiella pneumoniae*, *Escherichia coli*, *Staphylococcus aureus* and *Pseudomonas aeruginosa* were used. The suspension test was used with contact time of 1, 5 and 10 min with starting concentration of 10^5^ bacterial colony forming unit per milliliters (CFU/mL). The log_10_ reduction value at specified bacteria-disinfectant contact time was used to assess the efficacy of 0.5%NaDCC and 4.8% chloroxylenol in-use and freshly prepared solutions. In-use 0.5%NaDCC demonstrated poor log reduction (˂ 5log) against MDR-clinical isolates. Freshly laboratory prepared 0.5% NaDCC had 100% microbial reduction at 1, 5 and 10 min of both ATCC and MDR strains up to 48 h after preparation when compared with freshly prepared 4.8% chloroxylenol (˂ 5log). Freshly, prepared 0.5% NaDCC should be used in health-care facilities for effective disinfection practices.

## Introduction

Disinfectants are important components of infections control intervention strategies in health-care facilities in preventing the emergence of healthcare associated infections (HCAIs)^1^. These chemicals are applied to eliminate the contamination of bacteria, viruses, and fungi present on the hospital environments such as patients’ beds, side tables, trolley and benches for the purpose of preventing HCAIs^1,2^.

The majority of multidrug resistant (MDR) bacteria are also resistant to commonly used disinfectants^3,4^. This is because, similar mobile genetic elements (MGEs) particularly plasmid which harbors genes encoding for resistance to multiple antibiotics also carrying genes encoding for resistance genes towards disinfectants^5^. The disinfection process can be affected by different variables such as temperature, contact time, pH, concentration of the disinfectant, bio-burden, organic materials and type of water used for dilution^5^. Therefore, in-use disinfectants should be evaluated to ensure their effectiveness, due to the fact that ineffectiveness of disinfectant can result to HCAIs outbreak^6–9^. In the neonatal units of the Bugando Medical Centre in Mwanza, bloodstream HCAIs have been associated with mortality of up to 19%^10,11^. In addition, these outbreak infections have been found to be due to pathogens contaminating the hospital environment with MDR *K. pneumoniae* being predominantly isolated^12^, necessitating this study to assess the efficacy of disinfectants from neonatal units.

Nevertheless, the link between disinfectant efficacy and MDR pathogens has not been well studied in our setting. Lack of this information limit the recommendations for best disinfection practices or appropriate disinfectant concentration which is efficacious. Therefore, this study was designed to assess the efficacy of 0.5% sodium dichloroisocyanurate and 4.8% chloroxylenol on control (ATCC) strains and their respective MDR clinical strains of *Pseudomonas aeruginosa, Escherichia coli, Klebsiella pneumoniae* and *Staphylococcus aureus* causing neonatal sepsis at a zonal referral hospital in Mwanza, Tanzania.

## Methods

### Study design, duration, and setting

This was a cross-sectional hospital-based study which was conducted between June and August 2022. Samples of the in-use disinfectants were collected from neonatal units of the Bugando Medical Centre (BMC) which is a tertiary referral hospital in the Lake Zone of Tanzania. Samples were processed in Microbiology Research Laboratory of the Catholic University of Health and Allied Sciences (CUHAS).

### Recovery of bacteria strains for testing

#### Source of bacteria for this study

A total of 4 bacterial species (*Klebsiella pneumoniae*, *Escherichia coli*, *Staphylococcus aureus* and *Pseudomonas aeruginosa*) isolated from clinical sample (blood) and confirmed to be multidrug resistant were used in this study. These bacteria were resistant to at least one agent in three or more chemical classes of antibiotic^12^.

ATCC strains of *E. coli* (ATCC 25922)*, K. pneumoniae* (ATCC 43816), *S. aureus* (ATCC 25923) and *P. aeruginosa* (ATCC 27853) and their respective MDR strains (*E. coli*, *K. pneumoniae*, *S. aureus* and *P. aeruginosa*) were recovered from − 80 °C freezer by sub-culture on MacConkey agar (MCA; HiMedia, Mumbai, India) and 5% sheep blood agar (SBA; HiMedia, Mumbai, India) and incubated in ambient air at 35 ± 2 °C for 24 h. The MDR strains were isolated from blood samples collected from neonates admitted in the BMC neonatal units between December 2018 and April 2019^12^.

### Sample collection of disinfectants available in neonatal units

In-use and in stock sodium dichloroisocyanurate (0.5% NaDCC, Scientific Laboratory Supplies, United Kingdom) and stock chloroxylenol (Planitol, Sri Balaji Pharma Ltd, Dar es Salaam, Tanzania) available in the neonatal units were sampled to test their antimicrobial efficacy against selected bacterial strains. In-use sample in neonatal unit, was prepared by dissolving 40 tables of 2.5 g NaDCC (4.72 g weight of tablet) in 10 L of tap water and stored in reusable plastic container. The solution was supposed to be within 24 h of preparation and the standard operating procedures require the solution to be prepared fresh every day.

Fifty (50 mL) of each disinfectant was collected in sterile Falcon 50 mL conical tubes (Corning; Arizona, United States). Samples of in-use disinfectants were collected twice a day, in the morning between 0800 and 0900 h and evening between 1600 and 1700 h for consecutive 14 days, with intervention at day three (instruction to prepare fresh everyday). This was done to examine the efficacy of the presumed freshly prepared 0.5% sodium dichloroisocyanurate (morning sample) and aged 0.5% sodium dichloroisocyanurate (evening sample). Whereas, in-stock disinfectants (2.5 g sodium dichloroisocyanurate and 4.8% chloroxylenol) were collected once and freshly prepared in the laboratory as recommended by the National Infection and Prevention Control (IPC) guidelines^13^.

### Laboratory preparation of disinfectants prior to testing of antimicrobial efficacy

Sample of the in-use disinfectant were directly tested for efficacy against selected bacterial strains while samples of in-stock disinfectant were reconstituted to obtain 0.5% sodium dichloroisocyanurate (NaDCC) and 1:20 chloroxylenol. Briefly, to make 0.5% of NaDCC, 4 tablets of NaDCC were dissolved in 1 L of tap water as documented previously^13^. The NaDCC was stored in closed plastic container for 3 days and tested each day to see if there is a decrease in efficacy. Whereas, to make 1:20 of chloroxylenol 4.8%, 2 mL of chloroxylenol were diluted in 40 mL of tap water^14^.

### Cleaning practice in neonatology ward

In routine practices, cleaning include daily wiping of the surfaces (tables, chairs and floor, sinks) and emptying waste bins twice a day. Detergent and chloroxylenol are used in cleaning the surfaces and NaDCC in cleaning the reusable non-autoclavable equipment like oxygen musk and in case there is a spillage. Dusting was done twice per week using chloroxylenol (Dettol).

### Suspension test to determine the antibacterial efficacy of in-use and in-stock disinfectants against selected bacteria strains

Suspensions of selected bacterial strains were prepared in the sterile 0.85% saline with turbidity equivalent to 0.5 McFarland standard solution (Remel Lenexa, Kansas US). This suspension was further diluted to obtain 10^5^ colony forming unit (CFU)/mL and used in this study. Bacterial suspensions were quantitatively inoculated on 5% sheep blood agar (SBA; HiMedia, Mumbai, India) plates and incubated at 35 ± 2 °C for 24 h to determine the initial growth before mixing with disinfectants. 1 mL of each bacterial suspension was mixed with 1 mL of each disinfectant sample in a separate sterile tube (Borosil Test tube; Mumbai, India), mixed gently and allowed to stand (contact time) for 1, 5 and 10 min before being quantitatively inoculated on BA plates. Prior to inoculation of SBA plates, 2 mL of neutralizing solution (Scharlau microbiology, Eur. Pharm. Spain) was added to stop the activity of disinfectant, then 1 µL of the mixture was quantitatively plated on SBA plate which was incubated in ambient air at 35 ± 2 °C for 24 h. The experiment was conducted in triplicate and mean values were used. The difference in growth between initial and final cultures (growths without and with disinfectants) was used assess a log reduction value of the disinfectants. The efficacy was determined based on the guidelines by the Environment Protection Agency (EPA) whereby a ≥ 5 log10 (99.999%) decrease of bacteria in less than 10 min was considered efficacious^15^.

### Quality control

*Escherichia coli* ATCC 25922 and *Staphylococcus aureus* ATCC 25923 were used as control strains to check for the performance quality of media and incubations conditions. Moreover, to ensure that sodium dichloroisocyanurate and chloroxylenol samples were sterile before the experiment, 1 µL of each of the disinfectants were inoculated on SBA plates and incubated at 35 ± 2 °C for 24 h.

### Data analysis

Data was analyzed by looking on log reduction using the equation, number of viable bacteria before addition of disinfectant (A) minus number of viable bacteria after addition of disinfectant(B). The equation was simplified as, Log Reduction = log_10_ (A/B).

Log reduction was defined as relative number of bacteria that eliminated after addition of disinfectant with specific exposure time (contact time). Thus, 1 log reduction means inactivating 90% of a target microbe, with a target microbial count being reduced by factor of 10.

### Ethics approval and permission

The protocol was approved by the joint Catholic University of Health and Allied Sciences and Bugando Medical Centre Research Ethics and Review Committee and assigned ethical clearance certificate number: CREC 591/2022. Permission to conduct this study was requested from hospital’s administration. Moreover, all methods in this study were performed in accordance with the relevant guidelines and regulations.

## Results

Twenty-eight (28) in-use 0.5% NaDCC samples were collected twice a day from neonatal wards and in-stock samples of 0.5% NaDCC and 4.8% were collected only once. In-use samples were all confirmed to be sterile i.e. no microbial growth of the cultured solution after 24 h of incubation.

The guidelines for preparation of 0.5% NaDCC was well documented and in place in neonatal units at this setting. However, in real practice, the units were preparing NaDCC with concentration of less than 0.5% by dissolving 4 tabs of 2.5 g NaDCC in 10 L of water resulting to 0.05% NaDCC solution.

### CFU/mL comparison between ATCC and MDR isolates for in-use sodium dichloroisocyanurate collected daily for 14 days.

The significant growth was observed at 1 and 5 min contact time for all ATCC strains in the first 3 days of collection. With the exception of *Pseudomonas aeruginosa* ATCC strain which had significant growth at 10 min, all other had no growth at 10 min on day 1. All clinical strains had significant growth at 1, 5 and 10 min with increase of growth from day 1 to day 3 (Table 1). Similar results were observed for in-use disinfectants collected in the morning and evening on the same day. After intervention at neonatal unit, no growth was observed for both ATCC and clinical MDR strain at contact time of 1, 5 and 10 min of the freshly prepared solution from day 4 to day 14.

**Table 1:** CFU/mL comparison between ATCC and MDR isolates for in-use sodium dichloroisocyanurate collected daily for 14 days.

Day-contact time	Organism mean CFU/mL of three experiments							
	ATCCKP	MDRKP	ATCCEC	MDREC	ATCCSA	MRSA	ATCCPA	MDRPA
Day 1								
0 min	6.44 × 10^5^	6.60 × 10^5^	1.82 × 10^5^	1.96 × 10^5^	2.73 × 10^5^	2.88 × 10^5^	7.13 × 10^5^	7.00 × 10^5^
1 min	5.00 × 10^5^	6.00 × 10^5^	1.62 × 10^5^	1.75 × 10^5^	2.9 × 10^4^	8.4 × 10^4^	2.75 × 10^5^	4.21 × 10^5^
5 min	4.0 × 10^4^	5.0 × 10^4^	1.4 × 10^4^	1.6 × 10^4^	1.2 × 10^3^	3.2 × 10^4^	5.2 × 10^4^	2.51 × 10^5^
10 min	0 × 10^0^	4.9 × 10^3^	0 × 10^0^	2.0 × 10^3^	0 × 10^0^	2.0 × 10^3^	5.0 × 10^3^	8.2 × 10^4^
Day 2								
0 min	2.38 × 10^5^	2.70 × 10^5^	1.87 × 10^5^	1.68 × 10^5^	4.50 × 10^5^	3.78 × 10^5^	4.32 × 10^5^	4.76 × 10^5^
1 min	1.51 × 10^5^	2.10 × 10^5^	1.23 × 10^5^	1.48 × 10^5^	2.81 × 10^5^	2.05 × 10^5^	3.42 × 10^5^	3.61 × 10^5^
5 min	2.7 × 10^4^	1.47 × 10^5^	1.3 × 10^4^	1.18 × 10^5^	6.2 × 10^4^	8.4 × 10^4^	1.78 × 10^5^	2.91 × 10^5^
10 min	4.0 × 10^3^	1.4 × 10^4^	3.0 × 10^3^	1.9 × 10^4^	2.0 × 10^3^	4.8 × 10^4^	9.6 × 10^4^	1.78 × 10^5^
Day 3								
0 min	3.19 × 10^5^	2.98 × 10^5^	6.76 × 10^5^	4.51 × 10^5^	6.51 × 10^5^	4.68 × 10^5^	5.18 × 10^5^	4.58 × 10^5^
1 min	2.76 × 10^5^	2.71 × 10^5^	3.72 × 10^5^	4.21 × 10^5^	3.42 × 10^5^	3.81 × 10^5^	4.85 × 10^5^	4.28 × 10^5^
5 min	1.40 × 10^5^	1.41 × 10^5^	2.55 × 10^5^	2.81 × 10^5^	2.39 × 10^5^	1.74 × 10^5^	2.80 × 10^5^	3.39 × 10^5^
10 min	4.2 × 10^4^	1.10 × 10^5^	8.3 × 10^4^	1.69 × 10^5^	5.5 × 10^4^	8.8 × 10^4^	1.85 × 10^5^	1.98 × 10^5^

*ATCC* American Type Culture Collection, *MDR* multidrug resistant, *KP* *Klebsiella pneumoniae*, *EC* *Escherichia coli*, *MRSA *methicillin resistant *Staphylococcus aureus*, *PA Pseudomonas aeruginosa.* 0 min means before application of disinfectant.

### Log reduction of ATCC strains and Clinical isolates using laboratory reconstituted 0.5% NaDCC

For laboratory reconstituted 0.5% NaDCC, we observed 5 log reduction (99.999%) of both ATCC and MDR strains after 1, 5 and 10 min of organism-disinfectant contact time within 2 days of use. In day 3, we observed a decreased efficacy of the laboratory reconstituted 0.5% NaDCC for 1 log reduction (90%), 2 log reduction (99%) and 5 log reduction (99.999%) at 1, 5 and 10 min, respectively (Table 2) with MDR *Pseudomonas aureginosa* demonstrating poor efficacy at 10 min contact time.

**Table 2:** Log reduction of standard strain and clinical isolates using freshly laboratory prepared NaDCC.

Days	Strain tested	CFU/mL before addition of NaDCC	Contact time and log reduction		
			1 min, n (%)	5 min, n (%)	10 min, n (%)
Day 1	ATCC KP	2.15 × 10^5^	5.33 (100)	5.33 (100)	5.33 (100)
	MDR KP	1.55 × 10^5^	5.19 (100)	5.19 (100)	5.19 (100)
	ATCC EC	2.10 × 10^5^	5.32 (100)	5.32 (100)	5.32 (100)
	MDR EC	3.66 × 10^5^	5.56 (100)	5.56 (100)	5.56 (100)
	ATCC SA	5.04 × 10^5^	5.70 (100)	5.70 (100)	5.70 (100)
	MRSA	4.90 × 10^5^	5.69 (100)	5.69 (100)	5.69 (100)
	ATCC PA	4.80 × 10^5^	5.68 (100)	5.68 (100)	5.68 (100)
	MDR PA	5.40 × 10^5^	5.73 (100)	5.73 (100)	5.73 (100)
Day 2	ATCC KP	3.08 × 10^5^	5.48 (100)	5.48 (100)	5.48 (100)
	MDR KP	2.40 × 10^5^	5.38 (100)	5.38 (100)	5.38 (100)
	ATCC EC	3.53 × 10^5^	5.55 (100)	5.55 (100)	5.55 (100)
	MDR EC	3.04 × 10^5^	5.48 (100)	5.48 (100)	5.48 (100)
	ATCC SA	3.42 × 10^5^	5.53 (100)	5.53 (100)	5.53 (100)
	MRSA	3.25 × 10^5^	5.51 (100)	5.51 (100)	5.51 (100)
	ATCC PA	2.21 × 10^5^	5.34 (100)	5.34 (100)	5.34 (100)
	MDR PA	2.08 × 10^5^	5.32 (100)	5.32 (100)	5.32 (100)
Day 3	ATCC KP	3.41 × 10^5^	1.15 (92.9)	5.53 (100)	5.53 (100)
	MDR KP	3.12 × 10^5^	0.96 (89.1)	1.45 (96.5)	5.49 (100)
	ATCC EC	3.02 × 10^5^	1.53 (97)	5.48 (100)	5.48 (100)
	MDREC	3.19 × 10^5^	0.85 (85.9)	1.66 (97.8)	5.50 (100)
	ATCC SA	2.04 × 10^5^	1.41 (96.1)	5.31 (100)	5.31 (100)
	MRSA	1.25 × 10^5^	0.87 (86.4)	1.39 (96)	5.09 (100)
	ATCC PA	3.23 × 10^5^	1.11 (96.3)	1.55 (97.2)	5.51 (100)
	MDR PA	3.44 × 10^5^	0.59 (74.1)	1.39 (95.9)	1.58 (97.4)

*ATCC* American Type Culture Collection, *MDR* multidrug resistant, *KP* *Klebsiella pneumoniae*, *EC* *Escherichia coli*, *MRSA *methicillin resistant *Staphylococcus aureus*, *PA Pseudomonas aeruginosa.*

### Log reduction for ATCC and MDR on disinfection with laboratory reconstituted 1:20 chloroxylenol

For laboratory reconstituted 1:20 chloroxylenol, we observed no log reduction after 1 min of organism-disinfectant contact time. Five log reduction (99.999%) was observed after 10 min of organism-disinfectant contact time for ATCC strains of *K. pneumoniae*, *E. coli* and *S. aureus* with less than 1 log reduction (75%) for ATCC strains of *Pseudomonas aeruginosa*. Whereas, for all MDR strains, the efficacy ranged from 1.7% for *Pseudomonas aeruginosa* to 35.5% for MRSA for a minute contact time, 4.4–72.8% for 5 min contact time and 24.8–97.1% for 10 min contact time (Table 3).

**Table 3:** Log and percent reduction for ATCC and MDR on disinfection with chloroxylenol.

Strain tested	CFU/mL before addition of Chloroxylenol	Contact time and log reduction		
		1 min, n (%)	5 min, n (%)	10 min, n (%)
ATCC—KP	4.51 × 10^5^	0.34 (54.9)	0.68 (79.2)	5.65 (100)
MDR—KP	3.41 × 10^5^	0.07 (14.3)	0.26 (45.0)	0.54 (71.1)
ATCC—EC	2.32 × 10^5^	0.39 (60.0)	1.42 (96.2)	5.37 (100)
MDR—EC	2.60 × 10^5^	0.04 (8.9)	0.22 (40.3)	0.44 (63.9)
ATCC—SA	3.90 × 10^5^	0.63 (76.5)	1.64 (97.7)	5.59 (100)
MRSA	3.09 × 10^5^	0.19 (35.6)	0.57 (72.8)	1.54 (97.1)
ATCC—PA	3.95 × 10^5^	0.09 (19.5)	0.31 (51.0)	0.61 (75.4)
MDR—PA	4.05 × 10^5^	0.01 (1.7)	0.02 (4.4)	0.12 (24.8)

*ATCC* American Type Culture Collection, *MDR* multidrug resistant, *KP* *Klebsiella pneumoniae*, *EC* *Escherichia coli*, *MRSA* methicillin resistant *Staphylococcus aureus*, *PA Pseudomonas aeruginosa.*

## Discussion

Chlorine-based disinfectants are highly effective when freshly prepared, thus i.e. 99.999% bacterial reduction but it is efficacy decrease on standing. In this study the results show that MDR bacteria have low log reduction value towards in-use 0.5% NaDCC as compared to ATCC strains. However, on laboratory reconstituted 0.5% NaDCC, it has 5 log reduction (99.999%) for both ATCC and MDR strains. We also observed that a 0.5% NaDCC was more effective than 4.8% chloroxylenol for both ATCC strains and MDR isolates.

The MDR strains were more resistant to in-use 0.5% NaDCC than ATCC strains this may be attributed to the co-existence of disinfectants resistance genes and antibiotic resistance genes in the same cassettes of resistance genes as reported previously^16^. It was observed that among isolates tested, *Pseudomonas aeruginosa* was found to significantly resist disinfectants. This can be explained by the intrinsic resistance mechanisms i.e., biofilm formation and efflux pump by *Pseudomonas aeruginosa* that contributing to its resistance toward disinfectants^17–19^.

The laboratory reconstituted sodium dichloroisocyanurate had good log reduction for both ATCC strains and MDR isolates when compared with in-use 0.5% NaDCC and laboratory reconstituted 4.8% chloroxylenol. The 4.8% chloroxylenol had poor log reduction for all MDR strains and totally inactive to *Pseudomonas aeruginosa*. The results are similar to Ochie et al*.*^20^, who reported regrowth of bacteria after disinfection with chloroxylenol. The NaDCC is more effective due to the ability to release the HOCl and OCl- molecule on dissolution with water which act as oxidizing agent aiding in killing microorganisms by disrupting the membrane and oxidize the enzymatic activity inside the bacteria^21^. Chloroxylenol act by releasing hydroxyl (–OH) groups of the chloroxylenol molecule binds to certain proteins on the cell membrane of bacteria, and disrupts the membrane so as to allow the contents of the bacterial cell to leak out^22^.

This study also observed poor preparation of 0.5% NaDCC in neonatal units at this setting. Practically, the units were preparing 0.05% NaDCC solution instead of 0.5% NaDCC due to the fact that, they were dissolving 4 tabs of 2.5 g NaDCC each in 10 L of tap water. Technically, 4 tabs of NaDCC 2.5 g each should be dissolved within 1 L of water to make 0.5% NaDCC^13^. This could explain poor log reduction in 3 days of study before intervention. This indicates that neonatal units at this setting have poor IPC practices, that contributing to cross-transmission of pathogens between patients and hospital environment as well as outbreaks of HCAIs infections^23^. Furthermore, we observed that freshly laboratory prepared 0.5% NaDCC had good efficacy (99.999%) within 48 h with poor efficacy observed in day 3 for both ATCC and MDR strains underscoring the importance of using freshly prepared 0.5% NaDCC for the decontamination.

## Conclusion

Freshly prepared 0.5% sodium dichloroisocyanurate is more potent than 4.8% chloroxylenol and had good efficacy against both clinical isolates and standard strains with significant decrease in efficacy for in-use solutions and storage beyond 48 h especially for clinical isolates.

## Recommendation

There is a need to ensure chlorine-based disinfectants are freshly prepared and exposure time monitored as per manufacturer instructions to ensure effective decontamination of the hospital surfaces contaminated with clinical isolates especially MDR pathogens. The freshly prepared sodium dichloroisocyanurate is efficacious to *Pseudomonas aeruginosa*. Constant monitoring of the correct preparation and use of disinfectant in cleaning procedures on the surfaces, equipment and accessories is critical in order to reduce cross contamination and cross transamination of MDR pathogens.

## Data Availability

The datasets used and/or analysed during the current study are available from the corresponding author on reasonable request.

## Abbreviations

ATCC: American Type Culture Collection
BMC: Bugando Medical Centre
CFU/mL: Colony forming unit per milliliter
CUHAS: Catholic University of Health and Allied Sciences
EPA: Environmental Protection Agency
HCAIs: Healthcare associated infections
MCA: MacConkey agar
MDR: Multidrug resistant
MGEs: Mobile genetic elements
NaDCC: Sodium dichloroisocyanurate
SBA: Sheep blood agar
